# Bowel and bladder outcomes in patients with anorectal malformations and sacral agenesis: a retrospective cohort study

**DOI:** 10.1007/s00383-026-06471-x

**Published:** 2026-05-28

**Authors:** J. N. Theeuwes, C. M. C. de Beaufort, C. J. McDonald, D. P. Bakker, J. van Schuppen, C. F. Kuijper, O. E. Arguedas Flores, J. R. de Jong, M. E. B. Kremer, R. R. Gorter

**Affiliations:** 1 Department of Pediatric Surgery, Emma Children’s Hospital Amsterdam UMC, University of Amsterdam, Meibergdreef 9, 1105 AZ Amsterdam, The Netherlands; 2 Amsterdam Gastroenterology Endocrinology Metabolism Research Institute, Amsterdam, Netherlands; 3 Amsterdam Reproduction and Development Research Institute, Amsterdam, Netherlands; 4 Department of Pediatric Neurology, Amsterdam UMC, University of Amsterdam, Meibergdreef 9, Amsterdam, the Netherlands; 5 Department of Radiology and Nuclear Medicine, Amsterdam UMC, University of Amsterdam, Meibergdreef 9, Amsterdam, the Netherlands; 6 Department of Pediatric Urology, Emma Children’s Hospital Amsterdam UMC, University of Amsterdam, Meibergdreef 9, Amsterdam, the Netherlands

**Keywords:** Anorectal malformation, Sacral agenesis, Fecal incontinence, Neurogenic bladder, Transanal irrigation, Clean intermittent catheterization

## Abstract

**Purpose:**

Patients with anorectal malformation (ARM) and associated sacral agenesis (SA) are at increased risk of long-term bowel and bladder dysfunction, but outcome data to guide early counselling and follow-up remain limited. We aimed to evaluate bowel and bladder outcomes in patients with concurrent ARM and SA, with a focus on transanal irrigation (TAI) and clean intermittent catheterization (CIC) use.

**Methods:**

In this retrospective single-center cohort study, all patients with ARM and SA born between January 2000 and January 2024 were included. SA was diagnosed radiologically and classified using Pang’s classification. The primary outcome was TAI and/or CIC use. Secondary outcomes included fecal incontinence (≥ 4 years), urinary incontinence (≥ 5 years), and neurogenic bladder.

**Results:**

In total, 41 patients were included, with a median age at follow-up of 11.2 years. Of 33 patients ≥ 4 years, 22 (66.7%) used TAI during follow-up, primarily for fecal incontinence. Fecal incontinence was present in 26.3% of current TAI users versus 71.4% of non-users (*p* = .015). CIC was initiated in 18 of 41 patients (43.9%), mostly due to urodynamically confirmed neurogenic bladder. TAI and CIC use were associated with ARM complexity, but not with radiological SA severity.

**Conclusion:**

Patients with concurrent ARM and SA represent a high-risk group for long-term bowel and bladder dysfunction, and many require structured bowel and/or bladder management. These findings support anticipatory counselling and multidisciplinary follow-up.

**Supplementary Information:**

The online version contains supplementary material available at 10.1007/s00383-026-06471-x.

## Introduction

Anorectal malformations (ARM) are a spectrum of congenital conditions affecting approximately 1 to 3 in every 5000 live births [1]. They range from minor anorectal defects to complex cloacal malformations, classified according to the Krickenbeck criteria [2]. Almost all individuals with ARM require reconstructive surgery in early infancy [1, 3]. Approximately 60% of patients with ARM have associated congenital anomalies, either as part of the VACTERL association (Vertebral, Anorectal, Cardiac, Tracheo-Esophageal, Renal, and Limb anomalies) or as part of genetic syndromes or other conditions [4, 5]. One such associated condition is sacral agenesis (SA), also referred to as caudal regression syndrome, which is a rare condition occurring in approximately 1 to 5 per 100,000 live births in the general population [4, 6]. This condition results from failure of formation or regression of caudal spinal structures during secondary neurulation, leading to abnormal development of the lower osseous spine. The radiological presentation of SA is highly variable, ranging from isolated absence of the coccyx to extensive agenesis of the sacral and lumbar vertebrae, and may include abnormal spinal cord morphology and termination level [6].

Both ARM and SA individually predispose patients to bowel and bladder dysfunction due to anatomical and neurological factors [7–9]. In patients with an isolated ARM, defecatory disorders often exist despite optimal definitive reconstructive surgery, with patients experiencing either fecal incontinence or constipation [10]. Achieving optimal continence typically necessitates bowel management strategies, such as pharmacological treatment, enemas or transanal or antegrade irrigation (TAI, ACE), tailored to individual patients’ needs. Logically, management might become even more complex in patients with concurrent SA, as SA-related neurological deficits could further impair anorectal function, contributing to either fecal incontinence or constipation [11, 12]. Similarly, SA is also known to cause bladder dysfunction, necessitating interventions such as clean intermittent catheterization (CIC) [13]. However, bladder dysfunction is not exclusive to SA; even without spinal anomalies ARM patients may develop secondary bladder dysfunction due to constipation, surgical trauma to the neural networks or simply due to abnormalities in neural development and anatomy, which further complicates management strategies [14–16].

Despite the co-occurrence of ARM and SA, their combined impact on long-term bowel and bladder outcomes remains poorly understood. Yet these children likely represent a high-risk group for substantial long-term functional morbidity, often requiring structured bowel and/or bladder management. Better insight into these outcomes is therefore important for anticipatory counselling, realistic expectation setting, and multidisciplinary follow-up. Accordingly, this study aimed to assess bowel and bladder outcomes in patients with concurrent ARM and SA, with specific attention to the need for TAI and CIC.

## Methods

### Study design and patient population

A retrospective single-center cohort study was conducted at the Emma Children’s Hospital, Amsterdam University Medical Center (Amsterdam UMC), and was designed in accordance with the Strengthening the Reporting of Observational Studies in Epidemiology (STROBE) guidelines [17]. Patients were identified through a retrospectively constructed database comprising individuals with ARM born between January 2000 and January 2024. This database included patients born at Emma Children’s Hospital, Amsterdam UMC as well as those referred to the center after birth or later in childhood. For the purposes of this study, only patients diagnosed with ARM combined with SA were included. Patients were excluded if they were not followed in the outpatient clinic, died within 3 days of birth, no imaging was available to confirm the SA diagnosis, or if parental objection to use of data was raised.

### Ethics

The medical ethics committee classified this study as non-WMO (ref. no. W19_293 #19.350). Parents or legal guardians of all identified patients received written information with an option to object, and patients were excluded from the database if objection was raised.

### Imaging review and data collection

For patient selection, ARM patients in whom sacral or spinal abnormalities were reported on original imaging were selected for further evaluation. Subsequently, available sacral and spinal imaging was systematically re-evaluated by a pediatric radiologist (JvS) to confirm the diagnosis of SA and classify severity according to Pang’s system [6]. Sacral anatomy was assessed using all available imaging, including vertebral X-ray and MRI. All patients were followed within our specialized multidisciplinary ARM follow-up pathway, with close collaboration between pediatric surgery, pediatric urology, radiology, and other specialties depending on the patient’s clinical needs. Data were retrospectively extracted from electronic medical records by one researcher (JT). Bowel and bladder outcomes, as well as interventions such as TAI and CIC, were recorded based on routine clinical documentation by the treating multidisciplinary team, including both inpatient and outpatient notes. In case of unclear or inconsistent reporting, clarification was obtained through consultation with the treating pediatric surgeon or urologist (RG, OAF). Follow-up was defined from birth until the most recent hospital visit, with data collected through January 2025. If imaging was unavailable or incomplete, this was recorded as missing.

### Definitions

ARM subtypes were classified according to the Krickenbeck classification with major clinical groups and more rare or regional variants[2]. To enable statistical analysis, ARM subtypes were grouped based upon expected severity into three categories: simple (perineal fistula, anal stenosis), intermediate (vestibular fistula, rectourethral fistula, no fistula), and complex (rectovesical fistula, cloacal anomalies, and other rare/regional variants). VACTERL association was defined according to the EUROCAT classification, using the STRICT-VACTERL definition, which requires three or more major anomalies in different VACTERL organ systems [18]. SA was defined as any degree of sacrococcygeal bony agenesis. Its severity was classified according to Pang’s system, which categorizes SA into five types based on the extent of bony defects (Table 1) [6]. As Pang’s classification system incorporates both the morphology and level of spinal cord termination, the conus medullaris was assessed on ultrasound and categorized in group 1, group 2, or normal (Table 2) [19]. Within group 2, it was separately noted whether radiological findings were suspicious for a tethered cord.

**Table 1:** Pang’s classification of SA

Type I	Total sacral agenesis with some lumbar vertebrae missing
Type II	Total sacral agenesis with preserved lumbar spine
Type III	Subtotal, where only some sacral segments are missing
Type IV	Hemivertebra configurations (unilateral or bilateral)
Type V	Coccygeal agenesis, either total or partial.

**Table 2:** Pang’s classification of conus medullaris termination

Group 1	Blunted or club-shaped termination at or above T12-L1 level
Group 2	Tapered or tethered termination below lower border of L1 body
Normal	Termination around middle third of L1 vertebra

Constipation and fecal incontinence were defined according to the Rome IV criteria for pediatric functional gastrointestinal disorders, based on clinical history and documentation in the medical record [20]. Bowel outcomes were assessed from age ≥ 4 years (with < 4 years included exploratorily for early TAI), as fecal continence is not expected before this age [20]. Bladder function outcomes, specifically urinary continence status and history of UTIs, were recorded when documented in the medical record and were interpreted in line with International Children’s Continence Society terminology where applicable [21]. A neurogenic bladder was defined as discoordination of the bladder sphincter and detrusor either with or without under- or overactivity [22]. This diagnosis was confirmed by urodynamic testing in which filling and emptying phases of the bladder were assessed in combination with bladder pressure measurements [21]. Bladder outcomes were assessed in patients of all ages, but urinary incontinence was only analyzed in those aged ≥ 5 years, because reliable toilet training is typically expected at this age [21].

### Outcomes

Primary outcome was the number of patients (%) requiring TAI (assessed in those aged ≥ 4 years at follow-up) and/or CIC (assessed in all patients) during follow-up. Secondary outcomes were the prevalence of fecal incontinence in patients ≥ 4 years (TAI users vs. non-TAI users), the prevalence of urinary incontinence (daytime incontinence and enuresis) in patients ≥ 5 years (CIC users vs. non-CIC users), the prevalence of neurogenic bladder, use of pharmacological interventions and the prevalence of UTIs. In addition, associations between TAI and CIC use and ARM severity and SA severity were assessed. For descriptive comparison with ARM complexity, sacrococcygeal bony defects were additionally grouped as < 3 versus > 3 missing sacrococcygeal elements.

### Statistics

Statistical analysis was performed using IBM SPSS Statistics for Windows, Version 28 (IBM Corp., Armonk, NY, USA). Descriptive statistics were used to analyze baseline characteristics and clinical outcomes. Binary or categorical variables were reported as counts (n, %) and, where applicable, proportions (n/N, %). Continuous variables were presented as mean with standard deviation (SD) or median with interquartile range (IQR), as appropriate. Group differences in categorical outcomes were assessed using Fisher’s exact test, as small cell counts were expected. Univariable analyses were performed to explore associations between ARM and SA severity, and treatment outcomes, including the use of TAI and CIC. Due to the small sample size and limited number of outcome events, multivariable logistic regression was not feasible. Statistical significance was defined as a p-value less than 0.05. Missing data were not imputed, analyses were performed on an available-case basis.

## Results

###  Study population

**Fig. 1:**
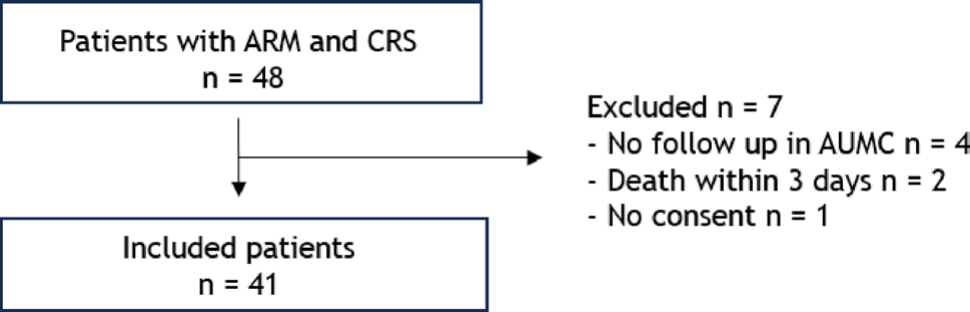
Flowchart for inclusion of patients

A total of 41 patients with ARM and SA were included in this study after application of the exclusion criteria (Fig. 1).

###  Patient characteristics

The study cohort showed a slight male predominance (*n* = 24, 58.5%), with a median age at follow-up of 11.2 years (IQR 6.0–16.5 years). ARM subtypes varied, with rectourethral fistula (*n* = 12, 29.3%) and cloacal malformation (*n* = 7, 17.1%) being the most common. Surgical correction was performed in 40 patients (97.6%), most frequently via posterior sagittal anorectoplasty (PSARP). Spinal and urinary tract imaging was part of the standard diagnostic work-up, with vertebral X-ray, spinal cord ultrasound and renal ultrasound performed in 39 (95.1%), 37 (90.2%) and 40 (97.6%) patients, respectively. One patient had total SA (*n* = 1, 2.4%), whereas partial SA was far more common (*n* = 34, 82.9%), with type III being the most prevalent subtype (*n* = 31, 75.6%). Abnormal spinal cord termination (group 1 or 2) was identified in 25 patients (61.0%). Additional characteristics are summarized in Table 3.

**Table 3:** Baseline characteristics (n=41)

			n (%), median (IQR)
Gender, male			24 (58.5)
Age at last follow-up, years			11.2 (6.0–16.5)
ARM type			
Simple			8 (19.5)
Rectoperineal fistula			5 (12.2)
Anal stenosis			3 (7.3)
Intermediate			17 (41.5)
Rectourethral fistula			12 (29.3)
Rectourethral fistula, unspecified			9 (22.0)
Rectobulbar fistula			2 (4.9)
Rectoprostatic fistula			1 (2.4)
Rectovestibular fistula			3 (7.3)
Imperforate anus without fistula			2 (4.9)
Complex			15 (36.6)
Rectovesical fistula			4 (9.8)
Cloacal malformation			7 (17.1)
Rare/regional variants			
Pouch colon			2 (4.9)
Rectovaginal fistula			2 (4.9)
Unknown			1 (2.4)
ARM operation type			
PSARP			19 (46.3)
ASARP			6 (14.6)
PSARPVUP			6 (14.6)
Pull through (details unknown)			2 (4.9)
Dilatation			2 (4.9)
Laparoscopic mini PSARP and pull through			2 (4.9)
TUM			1 (2.4)
Definitive stoma			1 (2.4)
None			1 (2.4)
Unknown			1 (2.4)
Vertebral imaging performed			
X–ray			39 (95.1)
Spinal cord ultrasound			37 (90.2)
MRI			26 (63.4)
SA – bony agenesis classification			
Type I			1 (2.4)
Type II			0 (0.0)
Type III			31 (75.6)
Type IV			3 (7.3)
Type V			6 (14.6)
SA – spinal cord termination			
Normal			16 (39.0)
Group 1:at or above T12-L1 level			8 (19.5)
Group 2: below lower border of L1 body			17 (41.5)
Tethered cord			10 (24.4)
Spinal cord anomaly^*^			14 (34.1)
Neurologic surgery performed^¶^			5 (12.2)
Urologic imaging performed			
Renal ultrasound			40 (97.6)
Urodynamic testing			30 (73.2)
Voiding cystourethrography			22 (53.7)
Urologic structural abnormalities^§^			20 (48.8)
VACTERL association			24 (58.5)
Syndrome^‡^			6 (14.6)

^*^Spinal cord anomalies included lipoma of filum terminale (n=4), teratoma (n=3), thickened filum terminale (n=3), thickened filum + intradural lipoma (n=2), lipomyelomeningocele (n=1), and intradural spinal lipoma (n=1)

^¶^Neurologic surgery consisted of excision sacrococcygeal teratoma (n=3), untethering tethered cord (n=1), transection of tight filum terminale (n=1)

^§^Urologic structural abnormalities included solitary kidney (n=7), hydronephrosis/dilated pelvicalyceal system (n=6), kidney atrophy (n=3), crossed fused renal ectopia (n=2), horseshoe kidney (n=1), and nephrocalcinosis (n=1)

^‡^Syndromes included Currarino (n=3), Pallister–Hall (n=1), Cat Eye syndrome (n=1), and terminal 8p deletion (n=1)

### Bowel control outcomes and TAI

In total, 33 of 41 patients (80.5%) were aged ≥ 4 years and therefore eligible for bowel function outcome analysis. Of these, 22 (66.7%) received TAI at some point during follow-up (Table 4). 19 of 22 patients (86.4%) were still using TAI at latest follow-up, whereas three (13.6%) discontinued the therapy. The most common indication for TAI was fecal incontinence in 15 patients (68.2%), with constipation (according to the Rome IV criteria) accounting for the remaining seven patients (31.8%). Median age at which TAI was initiated was 4.6 years (IQR 3.5–6.5 years). Overall, 4 of 33 patients (12.1%) achieved full fecal continence without the use of TAI. Patients using TAI at latest follow-up (*n* = 19) had significantly lower rates of fecal incontinence compared to patients not using TAI at follow-up (*n* = 14, including 3 patients who had discontinued TAI) (26.3% vs. 71.4%, *p* =.015) (Table 5). Among patients who did not use TAI, two patients with daily fecal incontinence were strongly advised to start TAI but their parents opted against it. In the subgroup of patients aged < 4 years (*n* = 8), one child started TAI at 2.0 years of age due to high stool frequency resulting in severe diaper rash, after which stool frequency decreased. Current TAI use was significantly associated with ARM complexity (*p* =.015), with 11 of 13 patients (84.6%) in the complex ARM group using TAI at latest follow-up. In contrast, no significant differences in TAI use were found according to SA severity, whether classified by degree of sacrococcygeal bony agenesis or by spinal cord termination level (*p* = 1.000 and *p* =.575, respectively). Full overview is provided in Supplementary Table 1.

**Table 4:** TAI in patients with ARM and SA (≥ 4 years of age, n=33)

		n (%), median (IQR)
TAI		
Yes		22 (66.7)
-Current*		19 (86.4)
-Stopped		3 (13.6)
No		11 (33.3)
Indication start TAI		
Fecal incontinence		15 (68.2)
Constipation		7 (31.8)
Age start TAI,years		4.6 (3.5–6.5)

*One patient transitioned from TAI to irrigation via CHAIT button. One patient performed bowel irrigation via their colostoma

**Table 5:** Bowel outcomes according to TAI use at latest follow-up

		Patients receiving TAI (n=19)	Patients not receiving TAI (n=14)	P value
Age, years, median (IQR)		11.8 (9.2–17.2.2.2)	13.7 (8.8–17.0.8.0)	
Fecal incontinence, n (%)		5 (26.3)	10 (71.4)	.015
Daily		1 (20.0)	3 (30.0)*	
Weekly		2 (40.0)	4 (40.0)	
Sporadically		1 (20.0)	3 (30.0)	
Unknown		1 (20.0)	0 (0.0)	
Pharmacologic therapy, n (%)		11 (57.9)	7 (50)	.732
Laxatives		10 (90.9)	5 (71.4)	
Enemas		0 (0.0)	2 (28.6)	
Enemas and laxatives		1 (9.1)	0 (0.0)	

*2 patients were strongly advised to start TAI, but declined therapy

### Urologic outcomes and CIC

CIC was initiated in 18 of 41 patients (43.9%). Urodynamically confirmed neurogenic bladder was identified in 17 patients, all of whom were started on CIC. Among patients diagnosed with neurogenic bladder, the majority had a complex ARM phenotype (9/16, 56.3%) and Pang type III sacral agenesis (12/17, 70.6%). Further details are provided in Supplementary Table 2. One additional patient started CIC because of postoperative urinary retention rather than neurogenic bladder. The median age at initiation was 2.1 months (IQR 0–13.4 months) (Table 6). At latest follow-up, 33 patients (80.5%) were aged ≥ 5 years and were therefore eligible for analysis of urinary incontinence. Among these, patients on CIC had a lower prevalence of urinary incontinence (1/9, 11.1%) than those not on CIC (6/24, 25.0%), although this difference was not statistically significant (*p* =.642) (Table 7). Urologic surgery was performed in ten patients (24.4%), including bladder augmentation with catheterizable channels and ureteral reimplantation. In the majority of these patients CIC was used both preoperatively (*n* = 7) and postoperatively (*n* = 9). The three patients not on CIC preoperatively underwent vesicostomy or cutaneous ureterostomy at birth or within the first month of life as a urinary diversion method to protect the kidneys. Current CIC use was not associated with SA severity as determined by degree of sacrococcygeal bony agenesis or by spinal cord termination level (*p* =.323 and *p* =.663, respectively). By contrast, current CIC use significantly increased with ARM complexity (*p* =.023), from 12.5% in simple to 53.3% in complex ARM. Full overview is provided in Supplementary Table 3.

**Table 6:** CIC and urologic operations in ARM and SA (n=41)

		n (%), median(IQR)
CIC		
Yes		18 (43.9)
-Current		11 (61.1)
-Stopped		7 (38.9)
No		23 (56.1)
Indication start CIC		
Neurogenic bladder		17 (94.4)
Postoperative urinary retention		1 (5.6)
Age start CIC, months		2.1 (0.0–13.4)
Urologic operation		10 (24.4)
Ureteral reimplantation		4 (40.0)
Bladder augmentation, Mitrofanoff without closure of bladder neck		2 (20.0)
Bladder augmentation, Mitrofanoffwith closure of bladder neck		1 (10.0)
Bladder augmentation, Monti with closure of bladder neck		1 (10.0)
Ureteral reimplantation and ureterostomy		1 (10.0)
Vesicostomy		1 (10.0)

**Table 7:** Bladder outcomes according to CIC use at latest follow-up

		Patients on CIC (n=11)	Patients not on CIC (n=30)	P value
Age, years, median (IQR)		11.2 (6.0–16.3.0.3)	10.9 (6.0–16.5.0.5)	
Urinary incontinence*, n (%)		1 (11.1)	6 (25.0)	.642
Enuresis		0 (0.0)	2 (33.3)	
Daytime and enuresis		1 (100.0)	4 (66.6)	
Excluded < 5 years		2	6	
Urinary tract infection, n (%)		10 (90.9)	8 (26.7)	**< **.001
Pharmacologic therapy, n (%)		8 (72.7)	5 (16.7)	.001
Anticholinergic agents		4 (50.0)	0 (0.0)	
Antibiotic prophylaxis		1 (12.5)	4 (80.0)	
Both		3 (37.5)	1 (20.0)	

*Urinary incontinence was only assessed in patients ≥5 years of age (n=33), as urinary incontinence still is physiological in younger children

### Combined TAI and CIC

Overall, 14 patients (34.1%) required combined use of both TAI and CIC to manage bowel and bladder function. Among them, nine (64.3%) achieved complete continence, with no fecal incontinence, daytime urinary incontinence or enuresis. In contrast, four (28.6%) patients continued to experience fecal incontinence, and one (7.1%) patient had persistent daytime urinary incontinence and enuresis despite this intensive regimen. Most patients requiring both TAI and CIC had a complex ARM phenotype (8/13, 61.5%) and Pang type III sacral agenesis (12/14, 85.7%). Further details regarding ARM and sacral/spinal characteristics are provided in Supplementary Table 4

### Distribution of sacrococcygeal bony defect severity according to ARM complexity

The distribution of sacrococcygeal bony defect severity across ARM severity groups is shown in Table 8. More extensive bony loss (> 3 missing elements) was observed across all ARM categories, without a clear predominance in complex ARM.

**Table 8:** Sacrococcygeal bony defect severity according to ARM complexity in patients ≥ 4 years (*n* = 33)

ARM type		< 3 missing sacrococcygeal elements, n (%)	> 3 missing sacrococcygeal elements n (%)
Simple		3 (50.0)	3 (50.0)
Intermediate		9 (69.2)	4 (30.8)
Complex		7 (53.8)	6 (46.2)
Unknown		0 (0.0)	1 (100.0)
Total		19 (57.6)	14 (42.4)

## Discussion

This study provides a detailed overview of functional bowel and bladder outcomes in a rare and clinically complex cohort of patients with both ARM and SA. Long-term invasive continence management was frequently required, with TAI initiated in 67% and CIC in 44% of patients, highlighting the substantial functional burden associated with concurrent ARM and SA.

In this study, only 12% of patients aged ≥ 4 years achieved full fecal continence without TAI at latest follow-up. This is substantially lower than generally reported for many isolated ARM subtypes (approximately 30% to 80%), even though continence rates vary strongly by ARM severity [23–25]. Despite the relatively high proportion of patients with complex ARMs in our cohort, continence outcomes are worse than would be expected based on ARM type alone [1, 26]. However, given the heterogeneity of the cohort and the lack of multivariable analysis, we were unable to disentangle the independent contributions of sacrococcygeal bony defects, spinal cord abnormalities, and ARM complexity to bowel dysfunction. This substantial functional burden was also reflected in the high use of TAI: it was initiated in two thirds of patients, and most users (86%) continued long-term, indicating tolerability and perceived effectiveness. Furthermore, consistent with prior studies, TAI users had a significantly lower prevalence of fecal incontinence than non-users, although the observational and retrospective design limits causal interpretation [27]. For clinical practice, these findings support early counselling that fecal continence is unlikely and that structured bowel management programs, including TAI, are frequently required in ARM patients with concomitant SA.

CIC was implemented in nearly half of patients, most commonly because of neurogenic bladder, as confirmed by urodynamic studies. This is consistent with earlier research indicating that greater ARM complexity and sacral or spinal cord anomalies are independently associated with an increased risk of lower urinary tract dysfunction, although robust prevalence data for neurogenic bladder in ARM remain limited [28]. An early study by Boemers et al. (1994) reported neurogenic bladder in 77% of patients with both ARM and sacral agenesis, compared to just 18% in those with isolated ARM [29]. The lower prevalence observed in our cohort potentially reflects a different diagnostic strategy, since urodynamic testing was performed primarily when results were expected to change management (e.g., persistent post-void residuals, urinary tract dilatation, previously diagnosed VUR, or recurrent UTIs). In contrast, Boemers et al. performed urodynamic testing routinely in all children, thereby also identifying subclinical abnormalities. Among CIC users, 53% required additional urologic surgery, suggesting that transurethral CIC alone was often insufficient for adequate bladder management and protection of kidney function [30]. Additional urologic surgery most commonly consisted of urinary diversion (vesicostomy or cutaneous ureterostomy) in younger children. In older patients, this more often involved ureteral reimplantation for reflux or bladder augmentation with a catheterizable channel in cases of progressive loss of bladder compliance or inability to catheterize transurethrally. In clinical care, these findings underscore the need for anticipatory counselling and close follow-up focused on kidney protection in patients diagnosed with a neurogenic bladder. In this context, the timing of initiation of these intensive interventions becomes an important clinical consideration. For CIC, indication and timing are relatively straightforward, because early initiation is primarily intended to protect the kidneys in patients at high risk of neurogenic bladder. In our cohort, the median age at initiation was 2 months, highlighting an already established proactive approach to kidney protection. In contrast, the optimal timing for TAI is less clearly defined, and existing guidelines do not specify an evidence-based age threshold. In clinical practice, TAI is usually introduced in a stepwise manner, often after less invasive measures have proven insufficient. Its timing is individualized and depends on symptom severity, response to conservative treatment, developmental readiness of the child, and family readiness to undertake a relatively intensive daily regimen [31]. In our cohort, TAI was usually initiated around early school age, aligning with expectations for social fecal continence in Dutch primary schools [32]. However, earlier initiation may be preferable in selected children with severe symptoms, recurrent dermatitis, or stool loss contributing to recurrent UTIs [33]. In our center, this individualized approach takes place within a structured multidisciplinary follow-up pathway from infancy into transition to adult care, with repeated assessment by pediatric surgery and specialized nursing staff and additional input from other disciplines when clinically indicated. This is particularly relevant in patients with concurrent ARM and SA, in whom bowel and bladder dysfunction may evolve over time. Interestingly, current use of either TAI or CIC was not statistically associated with radiological SA severity, whereas both interventions were used significantly more often in patients with complex ARM. Additional descriptive analysis showed that the extent of sacrococcygeal bony loss did not clearly increase with ARM complexity. This suggests that the greater use of TAI and CIC in patients with complex ARM cannot be explained by sacral bony severity alone, although associated sacral and spinal abnormalities likely remain important contributors to the overall functional burden in this cohort. Together, these findings underscore the difficulty of separating the respective contributions of ARM complexity, sacral/spinal abnormalities, and other associated anomalies in this retrospective cohort. This is in line with previous ARM studies showing that sacral anomalies and a low sacral ratio are associated with poorer continence outcomes, while more recent multivariable analyses suggest that ARM subtype may be a stronger independent predictor of fecal continence than sacral development itself [13, 24, 28]. These findings highlight the complexity of continence prognosis in patients with ARM and SA and underscore that important clinical questions remain, particularly regarding early identification of children who will later require intensive bowel and bladder management and the added value of functional assessment beyond radiology alone.

Strengths and limitations of this study must both be considered when interpreting the findings. A key strength is the large cohort size for such a rare and complex condition, allowing for meaningful descriptive analysis. With a median follow-up age of 11.2 years, this study captures a long follow-up period. Additionally, to our knowledge this is the first study to assess both bowel and bladder outcomes in patients with concurrent ARM and SA, providing a comprehensive overview of functional outcomes across conditions that are often studied separately. Although the substantial burden of dysfunction may not be entirely unexpected to clinicians involved in the long-term care of these patients, the present study helps to quantify this burden in a relatively large cohort for such rare conditions. However, limitations include the retrospective design and reliance on clinical documentation, which may introduce reporting bias or variability in outcome definitions. Nevertheless, we suspect that functional problems were more likely to be underestimated than overestimated. Second, the cohort was clinically heterogeneous, particularly with respect to underlying anatomical and neurological complexity, which may have contributed to variability in functional outcomes. This was especially relevant in patients with cloacal malformations and spinal cord teratoma, who were retained to reflect clinical tertiary care practice. Finally, the limited number of outcome events precluded the use of multivariable logistic regression, thereby reducing the ability to control for confounders and limiting generalizability. Given the rarity of these conditions, multicenter collaboration and prospective data collection will be important to improve the evidence base, refine prediction of management needs, and better understand long-term functional and psychosocial outcomes.

In conclusion, long-term invasive continence management is frequently required in patients with concurrent ARM and SA, with many patients initiating TAI and/or CIC based on persistent fecal incontinence and suspected or confirmed neurogenic bladder. These findings may help contextualize expectations regarding long-term bowel and bladder care and underline the importance of structured multidisciplinary follow-up, which is already routine practice in our center.

## Supplementary Information

Below is the link to the electronic supplementary material.

Supplementary material 1 (DOCX 18.7 kb)

## Data Availability

Requests for data sharing will be considered by the study steering group upon written request to the corresponding author.

## Abbreviations

ARM: Anorectal malformation
TAI: Transanal irrigation
ACE: Antegrade colonic enema
CIC: Clean intermittent catheterization
VACTERL: Vertebral, Anorectal, Cardiac, Tracheo-Esophageal, Renal, and Limb anomalies
