# Supplementary material for: Bowel and bladder outcomes in patients with anorectal malformations and sacral agenesis: a retrospective cohort study
Source: Pediatr Surg Int. 2026 May 28;42(1):244. doi: 10.1007/s00383-026-06471-x (PMC13219063; doi:10.1007/s00383-026-06471-x)
Supplement: Supplementary file 1 — Supplementary material 1 (DOCX 18.7 kb) [file 383_2026_6471_MOESM1_ESM.docx]

**Supplementary Material**

Supplementary Table 1. Current use of TAI in patients ≥ 4 years (n=33) by ARM severity, Pang classification and spinal cord termination level

|  | | TAI, n (%)  (n=19) | No TAI, n (%)  (n=14) | P value |
| --- | --- | --- | --- | --- |
| ARM severity* | |  |  |  |
|  | Simple | 1 (16.7) | 5 (83.3) |  |
|  | Intermediate | 7 (53.8) | 6 (46.2) |  |
|  | Complex | 11 (84.6) | 2 (15.4) | **.015** |
| Pang classification | |  |  |  |
|  | Type III | 15 (57.7) | 11 (42.3) |  |
|  | Type IV | 2 (66.7) | 1 (33.3) |  |
|  | Type V | 2 (50.0) | 2 (50.0) | 1.000 |
| Spinal cord termination | |  |  |  |
|  | Normal | 8 (50.0) | 8 (50.0) |  |
|  | Group 1 | 3 (50.0) | 3 (50.0) |  |
|  | Group 2 | 8 (72.7) | 3 (27.3) | .575 |

*One patient from no TAI group with unknown ARM type was excluded

Supplementary Table 2. ARM severity, Pang classification and spinal cord termination level in patients diagnosed with neurogenic bladder (n=17)

|  | | n (%) |
| --- | --- | --- |
| ARM severity* | |  |
|  | Simple | 1 (6.3) |
|  | Intermediate | 6 (37.5) |
|  | Complex | 9 (56.3) |
| Pang classification | |  |
|  | Type I | 1 (5.9) |
|  | Type III | 12 (70.6) |
|  | Type IV | 1 (5.9) |
|  | Type V | 3 (17.6) |
| Spinal cord termination | |  |
|  | Normal | 5 (29.4) |
|  | Group 1 | 5 (29.4) |
|  | Group 2 | 7 (41.1) |

*ARM type was unknown in one patient

Supplementary Table 3. Current use of CIC in patients (n=41) by ARM severity, Pang classification and spinal cord termination level

|  | | CIC, n (%)  (n=11) | No CIC, n (%)  (n=30) | P value |
| --- | --- | --- | --- | --- |
| ARM severity* | |  |  |  |
|  | Simple | 1 (12.5) | 7 (87.5) |  |
|  | Intermediate | 2 (11.8) | 15 (88.2) |  |
|  | Complex | 8 (53.3) | 7 (46.7) | **.023** |
| Pang classification | |  |  |  |
|  | Type I | 1 (100.0) | 0 (0.0) |  |
|  | Type III | 7 (22.6) | 24 (77.4) |  |
|  | Type IV | 1 (33.3) | 2 (66.7) |  |
|  | Type V | 2 (33.3) | 4 (66.7) | .323 |
| Spinal cord termination | |  |  |  |
|  | Normal | 3 (18.8) | 13 (81.3) |  |
|  | Group 1 | 2 (25.0) | 6 (75.0) |  |
|  | Group 2 | 6 (35.3) | 11 (64.7) | .663 |

*One patient from no CIC group with unknown ARM type was excluded

Supplementary Table 4. ARM severity, Pang classification and spinal cord termination level in patients requiring both TAI and CIC (n=14)

|  | | n (%) |
| --- | --- | --- |
| ARM severity* | |  |
|  | Simple | 1 (7.7) |
|  | Intermediate | 4 (30.8) |
|  | Complex | 8 (61.5) |
| Pang classification | |  |
|  | Type III | 12 (85.7) |
|  | Type IV | 1 (7.1) |
|  | Type V | 1 (7.1) |
| Spinal cord termination | |  |
|  | Normal | 5 (35.7) |
|  | Group 1 | 4 (28.6) |
|  | Group 2 | 5 (35.7) |

*ARM type was unknown in one patient
